# Editorial: Opportunities for PET imaging for the identification, staging, and monitoring of cancers

**DOI:** 10.3389/fonc.2023.1135928

**Published:** 2023-01-24

**Authors:** Jing Sun, Zhi Yuan Sun, Long Jiang Zhang

**Affiliations:** Department of Radiology, Jinling Hospital, Medical School of Nanjing University, Nanjing, China

**Keywords:** PET, cancer, identification, staging, monitoring

Cancer is one of the leading causes of death worldwide. Early diagnosis and treatment, as well as precise tumor staging are very important for the therapy choice and prognosis in oncology. PET can provide functional and metabolic information at the cellular and molecular level, allowing the early detection of cancer. Using molecular probes targeting specific tumor cells, PET enables ultra-early diagnosis and molecular typing of tumors. Combined with CT or MRI modality delivering anatomical information, the hybrid imaging modality (PET/CT or PET/MR) can provide important diagnostic information for oncology. The introduction of clinical PET/CT and PET/MR has led to a dramatic innovation in clinical research and patient management. The applications include diagnosis, staging and treatment response monitoring. Especially with the rapid development of new radiotracers, applications of multi-parameter functional MR imaging and artificial intelligence or radiomics technology, PET has become a very important tool in oncology.

## Identification

PET enables tumor imaging at the cellular/molecular level and early detection of cancer *via* visualization distribution of the specific and nonspecific radiotracer. Metabolism is higher in most malignant tumor cells than benign tumors, so radiotracers such as ^18^F-FDG, ^18^F/^11^C-CHO, and ^11^C-MET can be used for the differential diagnosis of benign and malignant tumors. Many functional MRI sequences, such as diffusion weighted imaging (DWI), perfusion weighted imaging (PWI) and magnetic resonance spectroscopy (MRS), can provide multidimensional functional information such as changes of water content and movement in tissue, microcirculation state and concentration of metabolites, to aid quantitative and qualitative diagnosis. These functional MRI sequences have been widely used in brain, lung, prostate and breast tumors. Diffusion-related parameters, such as mean diffusivity (MD) and apparent diffusion coefficient (ADC), reflect the restricted diffusion of water molecules in the tissue and are closely related to the cell density, intracellular matrix and number of organelles. Feng et al. found both ADC and MD values were weakly negatively correlated with the Ki-67 index, which is associated with the cell density, nucleocytoplasmic ratio and tumor invasive ability. Recent studies have confirmed that volume-based PET parameters, such as metabolic tumor volume (MTV) and total lesion glycolysis (TLG), reflect the biological information of tumors more comprehensively, especially in the assessment of lung cancer stage (1–3). Feng et al. found that SUV_max_, MTV and TLG values were significantly different between the squamous cell carcinoma group and adenocarcinoma group, which was very important in the identification of non-small cell lung cancer subtypes. Artificial intelligence and radiomics technologies can perform quantitative high-throughput image phenotypic analysis and identify important discriminant features to form effective radiomic features, that can be used effectively for disease detection, classification, prediction and prognosis evaluation. Wang et al. extracted radiomics features from^18^F-PSMA-1007-PET/CT imaging of primary prostate cancer to predict the tumor malignancy and clinical risk stratification. The study showed that PET/CT radiomic features alone had similar benefits as combined prostate specific antigen levels and metastatic status (4). Zhou et al. evaluated the predictive value in risk classification of the parameters of ^18^F-DCFPyL PSMA PET/CT. They found that prostate/muscle (P/M) ratio was the better parameter for risk classification of prostate cancer than SUV_max_.

## Staging

Combined the anatomical information provided by CT or MR imaging, PET/CT and PET/MR imaging enable accurate tumor staging. CT and MR imaging can provide anatomical details of the lesions including adjacent tissue invasion around tumors, even can show small distant metastasis, which can be helpful for more accurate TNM staging and has been of great clinical value especially in head and neck cancer (5, 6), breast cancer (7) and abdominal and pelvic tumors (8). In addition, PET can detect metastatic lymph node lesions which are small but clinically suspected with active FDG metabolism or high signal on DWI. The early detection of distant metastases can influence the tumor staging and the following treatment strategy (9, 10). Therefore, PET/CT and PET/MR can more accurately stage the tumor to provide more objective basis for the selection of individualized treatment.

## Monitoring and prediction

PET can more accurately monitor and predict the therapeutic response of tumor radiotherapy and chemotherapy and metabolic activity in the early stage of the lesion. Metabolic assessment by ^18^F FDG, measurement of mitotic activity by FLT, and application of new radiotracers such as amino acid tracers and their analogues can differentiate tumor recurrence from radiation necrosis. In addition, ^18^F-FET can also predict the prognosis of patients, and ^18^F-FDOPA PET can monitor tumor therapeutic response. For example, for pancreatic cancer patients who are not sensitive to chemotherapy and the RECIST criteria are not applicable to the evaluation of therapeutic efficacy, the metabolic information provided by PET/MR imaging can help identify early treatment response. Bevacizumab combined with chemotherapy has been approved by the United States Food and Drug Administration (US FDA) as a first-line treatment for locally unresectable advanced, recurrent, metastatic, non-squamous non-small cell lung cancer, however, who would benefit from this combination remains still unclear (11). Liu et al. combined^18^F-RGD PET/CT parameters with systemic immune factors to predict outcomes of patients with advanced non-small cell lung cancer receiving combined antiangiogenic treatment. They found ^18^F-RGD uptake on PET/CT and baseline serum inflammation biomarker could be used as predictive markers for combined antiangiogenic treatment. Similar results were reported by Hu et al.

In conclusion, PET, especially PET/CT and PET/MR imaging, has been widely used in oncology by providing both anatomical, functional and metabolic information at the cellular and molecular level. With the introduction of more tissue characterization techniques and multi-parameter MR imaging techniques, high-throughput image quantitative analysis methods such as artificial intelligence and radiomics, and the development of novel PET tracers, PET/CT and PET/MR would show a broader application prospective in the early diagnosis, staging, efficacy evaluation and prognosis prediction in oncology.

## Author contributions

JS: Writing-original draft preparation. ZS and LZ: Reviewing and Editing. All authors contributed to the article and approved the submitted version
